# Potential Cost Savings Associated with Targeted Substitution of Current Guideline-Concordant Inpatient Agents with Omadacycline for the Treatment of Adult Hospitalized Patients with Community-Acquired Bacterial Pneumonia at High Risk for *Clostridioides difficile* Infections: Results of Healthcare-Decision Analytic Model from the United States Hospital Perspective

**DOI:** 10.3390/antibiotics10101195

**Published:** 2021-10-01

**Authors:** Thomas Lodise, Mauricio Rodriguez, Surya Chitra, Kelly Wright, Nimish Patel

**Affiliations:** 1 Albany College of Pharmacy and Health Sciences, Albany, NY 12208, USA; 2 Paratek Pharmaceuticals, Inc., King of Prussia, PA 19406, USA; mrodriguez@sperotherapeutics.com (M.R.); surya.chitra@paratekpharma.com (S.C.); kelly.wright@paratekpharma.com (K.W.); 3 Skaggs School of Pharmacy & Pharmaceutical Sciences, University of California San Diego, La Jolla, CA 92093, USA; nipatel@health.ucsd.edu

**Keywords:** community-acquired pneumonia, antibiotics, omadacycline, *Clostridioides difficile* infection

## Abstract

Introduction: Approximately 3% of hospitalized patients with community-acquired bacterial pneumonia (CABP) develop healthcare-associated *Clostridioides difficile* infection (HCA-CDI). The validated Davis risk score (DRS) indicates that patients with a DRS ≥ 6 are at an increased risk of 30-day HCA-CDI. In the phase 3 OPTIC CABP study, 14% of CABP patients with DRS ≥ 6 who received moxifloxacin developed CDI vs. 0% for omadacycline. This study assessed the potential economic impact of substituting current guideline-concordant CABP inpatient treatments with omadacycline in hospitalized CABP patients with a DRS ≥ 6 across US hospitals. Methods: A deterministic healthcare-decision analytic model was developed. The model population was hospitalized adult CABP patients with a DRS ≥ 6 across US hospitals (100,000 patients). In the guideline-concordant arm, 14% of CABP patients with DRS ≥ 6 were assumed to develop an HCA-CDI, each costing USD 20,100. In the omadacycline arm, 5 days of therapy was calculated for the entire model population. Results: The use of omadacycline in place of guideline-concordant CABP inpatient treatments for CABP patients with DRS ≥ 6 was estimated to result in cost savings of USD 55.4 million annually across US hospitals. Conclusion: The findings of this simulated model suggest that prioritizing the use of omadacycline over current CABP treatments in hospitalized CABP with a DRS ≥ 6 may potentially reduce attributable HCA-CDI costs. The findings are not unique to omadacycline and could be applied to any antibiotic that confers a lower risk of HCA-CDI relative to current CABP inpatient treatments.

## 1. Introduction

Infections due to *Clostridioides difficile* (CDI), a common adverse effect associated with use of board-spectrum antibiotic therapy [1,2,3,4,5,6,7], is a leading cause of healthcare-associated infections. Such infections cause an estimated 2.24 cases per 1000 hospital admissions per year [8]. The occurrence of CDI places a substantial economic burden on the US healthcare system, resulting estimated annual attributable healthcare costs of nearly USD 5 billion in the USA [9]. It is also a significant source of lost revenue for many US hospitals as the additional healthcare expenditures associated with HCA-CDI often result in total hospital costs that exceed the amount reimbursed [10,11]. Due to the clinical and economic consequences associated with CDI, prevention of CDI is now a mandated national priority in the USA [12] and is part of the Centers for Medicare & Medicaid Services’ Hospital-Acquired Condition Reduction Program (HACRP) [13]. In recent years, multiple action plans and initiatives have been introduced to reduce CDI rates. These include antibiotic stewardship programs, national surveillance and action plans, improved testing methods, and enhanced infection control [14,15]. However, most CDI-risk reduction initiatives are aimed at secondary prevention. Given that use of broad-spectrum antibiotics, in large part, drive healthcare-associated CDI (HCA-CDI) rates [1,2,3,4,5,6], there is need to proactively use validated CDI risk stratification tools such as the Davis risk score (DRS) [3] to minimize the use of high CDI risk antibiotics [16], such as fluoroquinolones and ceftriaxone, in high-CDI-risk patients as a primary HCA-CDI prevention measure.

Community-acquired bacterial pneumonia (CABP) is a common infection type where there is a potential opportunity to risk stratify patients based on CDI risk and prioritize use of lower CDI risk antibiotics in high CDI risk patients. Data suggest that there are approximately 1 million CABP admissions per year in the United States of America [17] and approximately 3% of patients hospitalized with suspected or documented CABP develop CDI [18]. Despite their increased propensity to cause CDIs, especially in patients at an increased risk of developing CDI, third-generation cephalosporins and fluoroquinolones continue to be the preferred first-line agents for hospitalized patients with suspected or documented CABP [1,2,3,4,5,6,7,16,19]. In a recent real-world evidence study, hospitalized CABP patients who received ceftriaxone plus azithromycin, one of the mostly used guideline-concordant CABP treatment regimens, were at substantially higher odds of developing HCA-CDI relative to other treatments [20]. 

One potential alternative to the current guideline-concordant inpatient CABP treatments that has a lower CDI-risk potential is omadacycline. Omadacycline is approved by the FDA for the treatment of adult patients with CABP and has demonstrated a low propensity to induce CDI in preclinical and clinical studies relative to current guideline-concordant inpatient CABP agents [21,22,23,24,25]. In the phase 3 OPTIC study that compared omadacycline to moxifloxacin for adult hospitalized CABP patients, 2% of CABP patients who received moxifloxacin developed CDI versus 0% for omadacycline [21]. Among patients in the phase 3 OPTIC study with a Davis risk score (DRS) ≥ 6 [3] (one of the most well-described predictive indices for 30-day risk of HCA-CDI), 14% of CABP patients treated with moxifloxacin developed CDI compared with no patients in the omadacycline-treated group, despite balanced CDI risks between treatment groups [25]. To better understand the economic implications of targeted substitution of current guideline-concordant CABP inpatients treatments (i.e., fluoroquinolones and ceftriaxone) with omadacycline in adult CABP inpatients with a DRS ≥ 6 [3], a US decision analytic healthcare model from the hospital perspective was developed. 

## 2. Methods

### 2.1. Model Structure and Study Population

**Figure 1:**
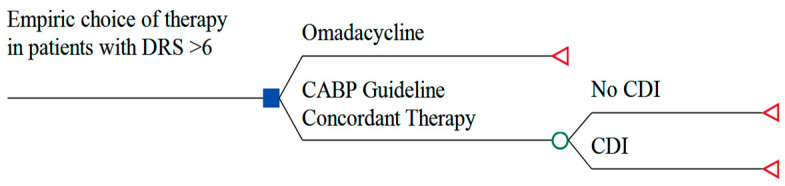
Decision analytic model from the US healthcare perspective. Abbreviations: DRS: Davis risk score; CABP: community acquired bacterial pneumonia; CDI: *Clostridioides difficile* infection.

A deterministic framework from the US hospital perspective was used to develop the conceptual healthcare-decision analytic model. The model population was hospitalized adult CABP patients with a DRS ≥ 6 (Figure 1). In the model, the use of guideline-concordant CABP inpatient treatments (i.e., fluoroquinolones and ceftriaxone) was replaced with omadacycline. The underlying model assumptions were: (1) CDI rates in hospitals can be reduced by limiting the use of broad-spectrum antibiotics such as fluoroquinolones and ceftriaxone [20,26,27,28], (2) omadacycline has a lower propensity to induce CDI relative to current guideline-concordant inpatient CABP treatments [1,2,3,4,5,6,7,21,22,23], and (3) omadacycline has the potential to minimize CDI events [21,25]. For the study population, it is estimated that 1 million adult patients in the United States of America are hospitalized with suspected or documented CABP annually [17] and 10% of CABP admissions occur in patients with a DRS ≥ 6 [3,21] (Table 1).

For the current guideline-concordant CABP treatment scenario, it is estimated that 14% of patients with a DRS ≥ 6 would develop an HCA-CDI. The 14% rate of CDI among patients with a DRS ≥ 6 in the guideline-concordant CABP treatment scenario was based on the observed CDI incidence among patients who received moxifloxacin in phase 3 OPTIC CABP study [21,25]. For the omadacycline scenario, no patients were assumed to develop CDI. This was based on the 0% CDI incidence among patients with a DRS ≥ 6 who received omadacycline in a phase 3 OPTIC CABP study [21,25]. 

### 2.2. Model Inputs and Assumptions

Only excess (i.e., attributable) hospital-related costs for the first HCA-CDI episode in CABP guideline-concordant group and drug acquisition costs in the omadacycline group were considered in the model. HCA-CDI was the only adverse event included in the model as the incidence of other adverse events was found to be similar between omadacycline and moxifloxacin in the OPTIC study. Initial hospital costs were assumed to be equal between treatment scenarios, regardless of treatment assignment. The treatment duration for both groups was assumed to be 5 days [34]. The drug acquisition costs in the guideline-concordant CABP treatment arm were assumed to be zero to align with the acquisition costs of levofloxacin and ceftriaxone, the most widely used agents for hospitalized patients with CABP in the United States of America. Omadacycline acquisition costs for 5 days of therapy were assumed to be USD 2260 based on the wholesale acquisition cost (WAC) for 5 days of IV omadacycline [32]. In the base case, the excess hospital inpatient cost associated for the development of an HCA-CDI were derived from a meta-analysis performed by the Agency for Healthcare Research and Quality (AHRQ), which estimated the attributable costs associated with an HCA-CDI to be USD 17,260 (95% confidence interval (CI): USD 9341–25,180) in 2015 USD [29]. When inflated to 2021 USD using the Consumer Price Index [33], the attributable hospital costs associated with HCA-CDIs are 20,100 (95% CI: 10,900–29,300). Hospital costs associated with recurrent HCA-CDI episodes were not considered in the model. 

### 2.3. Model Output and Analyses

Total attributable costs associated for each treatment scenario and incremental cost differences between treatment scenarios were determined for the base-case analysis. In the CABP guideline-concordant inpatient treatment arm, the attributable HCA-CDI costs for the 14,000 predicted CABP patients with CDI were calculated. In the omadacycline arm, the cost of 5 days of treatment for the estimated 100,000 CABP patients with DRS ≥ 6 was determined.

### 2.4. Parameter Sample Sensitivity Analyses

Second-order, probabilistic, parameter sample sensitivity analyses were performed to assess the effect of varying the attributable costs associated with an HCA-CDI. These variables were selected because they were the most influential variables within the structural model. A triangular distribution was applied to capture the variance surrounding this variable. The minimum and maximum values for the hospital costs associated with development of an HCA-CDI were set at USD 10,900 and USD 29,300, respectively [29]. These values were the 95% CI values for the attributable hospital cost of an HCA-CDI from the meta-analysis performed by the Agency for Healthcare Research and Quality (AHRQ); this meta-analysis estimated the additional hospital inpatient cost associated with HCA-CDI and other selected hospital-acquired conditions (HAC). A 5000-sample Monte Carlo simulation was performed to estimate mean (standard deviation), median (interquartile range and minimum/maximum) costs in the guideline-concordant CABP treatment arm and distribution of cost differences between treatment groups. 

### 2.5. Sensitivity Analyses

One-way sensitivity analyses were performed to determine the absolute difference in HCA-CDI rates per 100 treated CABP patients with a DRS ≥ 6 that still resulted in cost saving with omadacycline relative to the guideline-concordant standard of care agents (dominance threshold). Like the primary analysis, the only costs included in the guideline-concordant standard of care scenario were the attributable HCA-CDI costs among those who experienced HCA-CDI. The only costs included in the omadacycline scenario were the cost of 5 days of IV omadacycline for the 100 treated CABP patients with a DRS ≥ 6. The attributable HCA-CDI cost from the AHRQ meta-analysis of USD 20,100 [29] was used in the “based case” one-way sensitivity analysis. This estimated attributable cost value is consistent with estimated attributable hospital costs associated with an HCA-CDI inflated to 2021 US dollars by Zhang and colleagues (USD 27,891.90, 95% CI: USD 27,005.77–28,822.98) [35]. 

To capture the range of attributable costs associated with an HCA-CDI, the range of values identified in the literature were also evaluated. For the lower end, USD 10,860 (inflated to 2021 US dollars) was used as it represented the lower end of attributable costs for HCA-CDI by Gabriel and colleagues [30]. This lower bound is consistent with several studies that estimated the attributable costs associated with HCA-CDIs [36,37]. The upper end of attributable hospital-related HCA-CDI costs was based on meta-analysis by Zhang and colleagues, which reported that the average HCA-CDI-attributable cost per case across 42 individual studies was approximately USD 34,157 (90% CI: USD 33,134–35,180) in 2015 US dollars [31]. When inflated to 2021 US dollars, the attributable cost of an HCA-CDI was USD 39,700. This attributable HCA-CDI cost estimate reflected the upper end of attributable costs associated with HCA-CDI in the meta-analysis performed by the AHRQ (USD 37,659.63 in 2021 US dollars) [31]. This projected attributable cost of an HCA-CDI is also consistent with a recent real-world data analysis of healthcare resource utilization and direct medical costs associated with CDI by Feurstadt and colleagues. Here, the inpatient costs reported among CDI patients were USD 43,677 for patients with no recurrences [38]. 

## 3. Results

The total attributable costs associated for each treatment scenario and incremental cost differences between treatment scenarios that were determined in base-case analysis are shown in Table 2. In the guideline-concordant standard of care scenario, the cost of CDI in the projected 14,000 CABP patients with DRS ≥ 6 who developed CDI in the base-care analysis was estimated to be USD 281.4 million (14,000 CDI cases × USD 20,100 per case = 281.4 million). In the omadacycline scenario, the cost of 5 days of IV omadacycline therapy for the 100,000 projected CABP patients with DRS ≥ 6 was USD 226 million (100,000 patients × USD 2260 for 5 days of IV omadacycline = USD 226 million). Based on these estimated costs, the use of omadacycline in place of guideline-concordant CABP inpatient treatments for patients with a DRS ≥ 6 was estimated to result in cost savings of USD 55.4 million per year (USD 281.4 million − USD 226 million = USD 55.4 million) across US hospitals. The 5000-sample Monte Carlo Simulation analysis, in which the attributable HCA-CDI hospital costs were varied between 10,900 and 29,300 [31], demonstrated a median (interquartile range) cost saving of USD 55.4 (17.6–93.7) million across US hospitals (Table A1 in the Appendix A).

**Figure 2:**
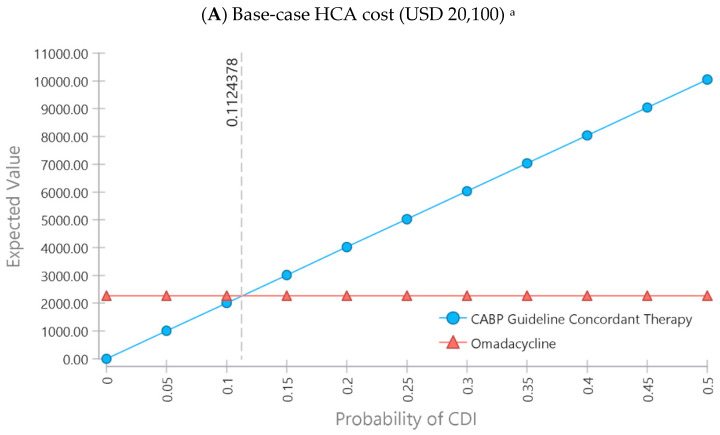

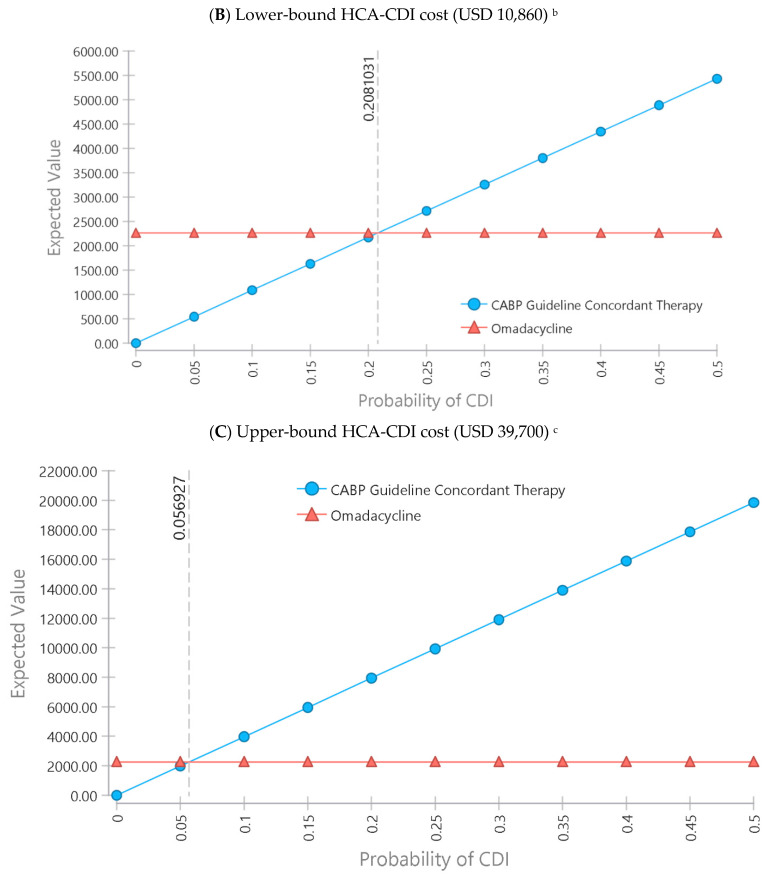
Cost-neutral HCA-CDI thresholds for omadacycline relative to guideline-concordant inpatient treatments for CABP patients with DRS ≥ 6 for (**A**) base-case HCA-CDI cost, (**B**) lower-bound HCA-CDI cost, and (**C**) upper-bound HCA-CDI cost. ^a^ Based on an estimated cost of USD 226,000 for 5 days of IV omadacycline treatment per 100 treated CABP patients with a DRS ≥ 6, the excess incidence of CDI in guideline-concordant patients would need to be 11.2% (11.24 cases per 100 patients × USD 20,100 per CDI case ≈USD 226,000) for omadacycline to be cost-neutral. ^b^ Based on an estimated cost of USD 226,000 for 5 days of IV omadacycline treatment per 100 treated CABP patients with a DRS ≥ 6, the excess incidence of CDI in guideline-concordant patients would need to be 21% (20.8 cases per 100 patients × USD 10,860 per CDI case ≈USD 226,000) for omadacycline to be cost-neutral. ^c^ Based on an estimated cost of USD 226,000 for 5 days of IV omadacycline treatment per 100 treated CABP patients with a DRS ≥ 6, the excess incidence of CDI in guideline-concordant patients would need to be 5.7% (5.7 cases per 100 patients × USD 39,700 per CDI case ≈USD 226,000) for omadacycline to be cost-neutral.

Results of the one-way sensitivity analyses to determine the absolute difference in attributable CDI rates between groups per 100-treated CABP patients with a DRS ≥ 6 that still resulted in cost saving with omadacycline is shown in Figure 2. For all the sensitivity analyses, the cost of 5 days of IV omadacycline treatment per 100 treated CABP patients with a DRS ≥ 6 was estimated to be USD 226,000. For the base case (the cost of each HCA-CDI case was 20,100 and cost of 5 days of IV omadacycline for 100 patients was USD 226,000), the excess incidence of CDI in guideline-concordant patients would need to be 11.2% (11.24 cases per 100 patients × USD 20,100 per CDI case ≈USD 226,000) for omadacycline to be cost-neutral (Figure 2A). At an attributable CDI hospital costs per case of USD 10,860, omadacycline had cost savings relative to guideline-concordant CABP treatments if there was a 21% excess incidence of HCA-CDI per 100 treated CABP patients with a DRS ≥ 6 (20.8 cases per 100 patients × USD 10,860 per CDI case ≈USD 226,000) (Figure 2B). At the upper end of the HCA-CDI cost per case range (USD 39,700), omadacycline had cost savings if the excess incidence of HCA-CDI in guideline-concordant patients was 5.7% (5.7 cases per 100 patients × USD 39,700 per CDI case ≈USD 226,000) (Figure 2C).

## 4. Discussion

There are (1) well-documented concerns with HCA-CDIs [8,10,11], (2) clinical and economic consequences associated with HCA-CDIs [9,10,11,29,30,31,35,36,37,38], and (3) a need for better proactive primary HCA-CDI prevention strategies [14,15,20]. As such, the intent of this study was to assess the economic impact of prioritizing the use of omadacycline (an agent approved for the treatment of adult patients CABP that has a documented lower propensity to cause CDI [21,22,23,24]), over other guideline-concordant inpatient therapies (fluoroquinolones and ceftriaxone) [19], in hospitalized CABP patients at high risk for CDI [3]. It is well documented that the use of guideline-concordant antibiotics such as fluoroquinolones and the advanced generation cephalosporins are associated with increased CDI risks [1,2,3,4,5,6,7,16,19] (especially in CABP patients at an increased risk of developing CDI [18,20,25]), and CDI rates can be reduced by limiting their use [26,27,28]. For this study, patients with a DRS ≥ 6 were considered high risk [3]. It is estimated that 10% of adult hospitalized CABP patients have a DRS ≥ 6 [3,21] and the typical profile of patients with a DRS ≥ 6 are those who are: (1) >55 years of age; (2) have ≥2 comorbidities, such as congestive heart failure, kidney disease, chronic pulmonary disease, peptic ulcer disease, liver disease cancer, dementia, diabetes, etc.; (3) currently receiving a protein pump inhibitor; and (4) received ≥1 high-risk CDI antibiotic in the past 30 days. Overall, the findings from the model suggested that prioritizing use of omadacycline over current guideline-concordant CABP treatments in hospitalized CABP patients with a DRS ≥ 6 has the potential to reduce CDI rates and substantially reduce attributable HCA-CDI hospital costs (Table 2). Based on model inputs, omadacycline would result in cost savings if it reduced 5–20 excess cases of CDI per 100 CABP-treated patients, depending on the attributable costs associated with an HCA-CDI, relative to the use of guideline-concordant CABP treatment in CABP inpatients with a DRS ≥ 6. Further study is needed to validate the model’s findings, but these results can serve as the basis of a proactive antibiotic stewardship initiative for healthcare institutions seeking to reduce hospital CDI rates and associated costs.

Several items should be noted when interpreting these findings. Findings are not unique to omadacycline and could be applied to any antibiotic that confers a lower risk of CDI relative to current CABP inpatient treatments [39,40]. We focused on attributable rather than total costs because many costs (e.g., hospitalization for initial treatment period, nursing time) would be the same regardless of the choice of antimicrobial agent. The conceptual models assumed that omadacycline did not lead to any cases of CDI. Thus, the one-way sensitivity analyses reflect the absolute difference in CDI rates between groups (i.e., excess CDI rate in guideline-concordant CABP treatment group) that still resulted in cost savings with omadacycline.

The major drivers of cost in the model were the attributable HCA-CDI hospital costs [29] and current WAC of 5 days of IV omadacycline [32]. For the primary analyses, we assumed that attributable HCA-CDI costs were USD 20,100. This attributable HCA-CDI cost value was identified in a meta-analysis that was performed by AHRQ to estimate the incremental inpatient financial costs associated with CDI and other selected HAC [29]. We selected this as the base-case attributable HCA-CDI cost since it being used by US federal agencies in tracking progress on improving patient safety and eliminating HAC. As attributable HCA-CDI costs vary across hospitals, the economic benefits associated with omadacycline presented in this study should be considered as an initial estimate of the potential value of omadacycline relative to other guideline-concordant CABP inpatient agents. We attempted to cover the range of potential attributable costs associated with HCA-CDI by performing a 5000-sample Monte Carlo simulation and several one-way sensitivity analyses. Healthcare systems should consider their local costs and their contracted omadacycline price when determining if the replacement of current guideline-concordant inpatient CABP therapies (i.e., fluoroquinolones and ceftriaxone) with an agent that has documented lower propensity to cause CDI, such as omadacycline, can improve the quality and efficiency of healthcare delivery within their system for patients at high risk for CDI. 

The reported value of omadacycline relative to guideline-concordant inpatient CABP therapies in CABP patients with DRS ≥ 6 were likely highly conservative and did not capture the full range of costs associated with the acquisition of an HCA-CDI. The model was limited to the attributable HCA-CDI hospital costs associated with the first CDI episode. Data suggest that the attributable healthcare may extend the first HCA-CDI hospitalization [7,9,10,11,35,36,38,41,42,43,44]. As such, results of the study should be viewed as conservative estimates of the potential cost savings associated with omadacycline relative to use of guideline-concordant inpatient CABP treatments in high-risk CDI patients with CABP. A key issue with the management of CDI is recurrent infection. Recurrent CDI occurs due to relapse or reinfection. Reports have shown that 18–25% of patients will experience at least one CDI recurrence [12,15,17,35,41,42,44]. In patients with ≥1 recurrence, the risk for subsequent recurrences increases to 45–65% [11,38,45]. A meta-analysis showed that that continuation of non-*C. difficile* antibiotics, advanced age, and use of antacid medications places patients at an increased risk for recurrence [46] and these risk factors are commonplace in CABP patients [19,21]. Attributable hospital costs associated with recurrent CDI are similar to those estimated for primary CDI episodes, and these costs should be considered when evaluating the study findings and potential economic benefits associated with omadacycline [9,10,11,35,36,37,38,41,44]. We also did not consider attributable HCA-CDI outpatient costs, which can also be substantial in patients with HCA-CDI [9,10,11,38]. Beyond incurring increased attributable costs, data also show that hospitals lose revenue among patients who develop a CDI during hospital and in most cases the margin between reimbursement and costs exceeds USD 5000 [10,11]. Finally, CDI is also part of the HAC reduction program, and hospitals are subject to having their Medicare payments reduced if their total HAC score is greater than the 75th percentile of all total HAC scores (i.e., the worst-performing quartile) [13]. 

There are also other aspects of these analyses that suggest the observed study results were highly conservative. The model was constructed from the hospital perspective and mortality was not incorporated into the model [47]. Each CDI episode is associated with a 5–10% increase in 30-day mortality and CDI-related mortality is upwards of 50% in high-risk patient populations [48,49]. The model also did not consider patient co-pays, satisfaction/quality of life/productivity, due to lack of comparator data on these endpoints between omadacycline and guideline-concordant CABP treatments. Data indicates that co-pays of Medicare patients, a high-risk group for primary and recurrent CDI, often exceeds USD 10,000 [11]. Data also indicates that HCA-CDI has considerable physical and psychologic consequences on patients and limits their ability to return to work and resume normal daily activities [50]. Finally, the model was limited to the excess costs associated with HCA-CDI and did not include other important consequences associated with reduction of third generation cephalosporins and fluroquinolones use, such as the reduction of extended spectrum beta-lactamase producing Gram-negatives and MRSA rates [51].

In conclusion, the hospital costs attributable to primary HCA-CDI and recurrent CDI are substantial [9,29,37,44]. It is critical that clinicians use proactive preventative strategies in high-risk patients with CABP to reduce the primary HCA-CDI burden. One way to minimize the occurrence of CDI is to prioritize the use of agents with a low propensity to cause CDI such as omadacycline over guideline-concordant CABP inpatient agents in high-risk CDI patients with CABP. The findings suggest that omadacycline has the potential to result in significant cost savings if its use is prioritized over current guideline-concordant treatments in CABP patients at high risk for CDI. As this was a modelling study, the findings need to be validated in clinical practice, but it can serve as the basis for a proactive CDI risk reduction antibiotic stewardship initiative.

## Figures and Tables

**Table 1:** Input parameters used in the deterministic healthcare-decision analytic model for base-case analyses.

Factor	Parameter
Number of CABP admissions per year in United States of America [17]	1,000,000
Percentage (number of patients) of hospitalized CABP patients with DRS ≥ 6 [3,21,25]	10% (n = 100,000)
Percentage (number of patients) of CABP patients with DRS ≥ 6 who developed CDI in the guideline-concordant standard of care scenario [21,25]	14% (n = 14,000)
Cost per episode of healthcare associated CDI in base-case analysis [29]	USD 20,100 (95% CI: 10,900–29,300)
Lower bound of cost per episode of healthcare associated CDI in one-way sensitivity analysis [30]	USD 10,860
Upper bound of cost per episode of healthcare associated CDI in one-way sensitivity analysis [31]	USD 39,700
Omadacycline wholesale acquisition cost for 5-day cost of IV therapy [32]	USD 2260

CABP, community-acquired bacterial pneumonia; CDI, *Clostridioides difficile* infection; DRS: Davis risk score; IV, intravenous. Costs are given in 2021 US dollars [33].

**Table 2:** Estimated attributable costs with use of guideline-concordant CABP inpatient treatment versus omadacycline in DRS ≥ 6 patients with CABP across United States hospitals in the base-case analysis.

Factor	Cost (USD Million)
Guideline-concordant standard of care scenario for CABP patients with DRS ≥ 6	281.4 ^a^
Cost of 5-day hospital treatment with omadacycline	226.0 ^b^
Cost saving from omadacycline scenario	55.4 ^c^

CABP, community-acquired bacterial pneumonia; CDI, *Clostridioides difficile* infection; DRS, Davis risk score. Costs are given in 2021 US dollars. ^a^ The estimated cost of CDI for the projected 14,000 CABP patients with DRS ≥ 6 in the guideline-concordant standard of care scenario who developed CDI (14,000 CDI cases × USD 20,100 per case = 281.4 million). ^b^ The estimated cost of 5 days of IV omadacycline therapy for the 100,000 projected CABP patients with DRS ≥ 6 in the omadacycline scenario (100,000 patients × USD 2260 for 5 days of IV omadacycline = USD 226 million). ^c^ Difference in cost between the omadacycline scenario and guideline-concordant standard of care scenario.

## Data Availability

Paratek Pharmaceuticals, Inc., has a commitment to ensure that access to clinical trial data is available to regulators, researchers, and trial participants, when permitted, feasible, and appropriate. Requests for deidentified patient-level data may be submitted to medinfo@paratekpharma.com for review.

## Appendix A

**Table A1:** Estimated Cost Savings Associated with Replacement of Guideline-Concordant CABP Inpatient Treatment with Omadacycline in Hospitalized Adult CABP Patients with DRS ≥ 6 Across United States Hospitals in 5000-sample Monte Carlo Simulation Analysis.

Standard-of-Care Scenario for CABP Patients with DRS ≥ 6	Cost Saving from Omadacycline Scenario Cost (USD Million)
Mean	55
Minimum	−68.1
Maximum	182.1
25th Percentile	17.6
Median	55.4
75th Percentile	93.7

HCA-CDI, Healthcare Associated-Clostridioides difficile infection; CABP, community-acquired bacterial pneumonia; HCA-CDI, Healthcare Associated-Clostridioides difficile infection; DRS: Davis risk score.
